# Unraveling the Genetics of Congenital Diaphragmatic Hernia: An Ongoing Challenge

**DOI:** 10.3389/fped.2021.800915

**Published:** 2022-02-03

**Authors:** Erwin Brosens, Nina C. J. Peters, Kim S. van Weelden, Charlotte Bendixen, Rutger W. W. Brouwer, Frank Sleutels, Hennie T. Bruggenwirth, Wilfred F. J. van Ijcken, Danielle C. M. Veenma, Suzan C. M. Cochius-Den Otter, Rene M. H. Wijnen, Alex J. Eggink, Marieke F. van Dooren, Heiko Martin Reutter, Robbert J. Rottier, J. Marco Schnater, Dick Tibboel, Annelies de Klein

**Affiliations:** ^1^Department of Clinical Genetics, Erasmus MC Sophia Children's Hospital, Rotterdam, Netherlands; ^2^Division of Obstetrics and Fetal Medicine, Department of Obstetrics and Gynecology, Erasmus MC Sophia Children's Hospital, Rotterdam, Netherlands; ^3^Department of Pediatric Surgery and Intensive Care, Erasmus MC Sophia Children's Hospital, Rotterdam, Netherlands; ^4^Unit of Pediatric Surgery, Department of General, Visceral, Vascular and Thoracic Surgery, University Hospital Bonn, Bonn, Germany; ^5^Center for Biomics, Erasmus MC Sophia Children's Hospital, Rotterdam, Netherlands; ^6^Department of Cell Biology, Erasmus MC Sophia Children's Hospital, Rotterdam, Netherlands; ^7^Department of Pediatrics, Erasmus MC Sophia Children's Hospital, Rotterdam, Netherlands; ^8^Institute of Human Genetics, University Hospital of Bonn, Bonn, Germany; ^9^Neonatology and Pediatric Intensive Care, Department of Pediatrics and Adolescent Medicine, University Hospital Erlangen, Erlangen, Germany

**Keywords:** foregut, genetics, development, counseling, diaphragm, hernia, discordant monozygotic twin, congenital

## Abstract

Congenital diaphragmatic hernia (CDH) is a congenital structural anomaly in which the diaphragm has not developed properly. It may occur either as an isolated anomaly or with additional anomalies. It is thought to be a multifactorial disease in which genetic factors could either substantially contribute to or directly result in the developmental defect. Patients with aneuploidies, pathogenic variants or *de novo* Copy Number Variations (CNVs) impacting specific genes and loci develop CDH typically in the form of a monogenetic syndrome. These patients often have other associated anatomical malformations. In patients without a known monogenetic syndrome, an increased genetic burden of *de novo* coding variants contributes to disease development. In early years, genetic evaluation was based on karyotyping and SNP-array. Today, genomes are commonly analyzed with next generation sequencing (NGS) based approaches. While more potential pathogenic variants are being detected, analysis of the data presents a bottleneck—largely due to the lack of full appreciation of the functional consequence and/or relevance of the detected variant. The exact heritability of CDH is still unknown. Damaging *de novo* alterations are associated with the more severe and complex phenotypes and worse clinical outcome. Phenotypic, genetic—and likely mechanistic—variability hampers ***individual*** patient diagnosis, short and long-term morbidity prediction and subsequent care strategies. Detailed phenotyping, clinical follow-up at regular intervals and detailed registries are needed to find associations between long-term morbidity, genetic alterations, and clinical parameters. Since CDH is a relatively rare disorder with only a few recurrent changes large cohorts of patients are needed to identify genetic associations. Retrospective whole genome sequencing of historical patient cohorts using will yield valuable data from which today's patients and parents will profit Trio whole genome sequencing has an excellent potential for future re-analysis and data-sharing increasing the chance to provide a genetic diagnosis and predict clinical prognosis. In this review, we explore the pitfalls and challenges in the analysis and interpretation of genetic information, present what is currently known and what still needs further study, and propose strategies to reap the benefits of genetic screening.

## Introduction

Congenital diaphragmatic hernia (CDH) [OMIM: 142340] has an estimated incidence of 1 in 1,750–5,880 live births (1–3) and is characterized by a defect of the diaphragm. This defect allows herniation of the abdominal organs into the thorax. CDH can be detected prenatally during first or second trimester ultrasounds in 50–68% of CDH pregnancies (4–7). Patients are often referred to a center of expertise with a specialized multidisciplinary team for prenatal assessment, prognostic and genetic counseling and care. CDH prevalence has slightly increased in the past years (3). Still, the mortality rates have decreased, probably due to better treatment strategies (8), although this decline is more pronounced in wealthier coutnries than in developing countries (9).

Most of what we know of human diaphragm development is based on descriptive and functional analyses of animal models. The diaphragm muscle develops initially from transient structures located at the top of the liver: the septum transversum, the pleuroperitoneal folds, the posthepatic mesenchymal plate, and the somites. Myoblast progenitors and other mesenchymal cells (10) in the developing pleuroperitoneal folds expand and migrate to the posthepatic mesenchymal plate. Vice versa, cells from the posthepatic mesenchymal plate migrate toward the pleuroperitoneal folds. Finally, the pleuroperitoneal folds fuse with the posthepatic mesenchymal plate between embryonic day (E) E12.5 and E13.5 (10, 11). When complete, this membrane separates the thoracic and abdominal cavity (E14.5). In CDH, this process is disrupted and the diaphragm will not fully close (12, 13). A more detailed description of diaphragm and CDH development can be found elsewhere in this issue (14).

Patients with aneuploidies, pathogenic single nucleotide variants, *de novo* Copy Number Variations (CNVs) (15–18) develop CDH, often in the form of a monogenetic syndrome and in combination with other anatomical malformations (2, 19). Here, we discuss what is currently known and inventoried what is necessary to provide optimal genetic counseling for the individual patients and their parents. We evaluate genetic outcome of a CDH cohort in the Erasmus MC-Sophia Children's Hospital, Rotterdam, the Netherlands, and propose strategies to reap the benefits of genetic screening.

## CDH Has Subtypes Based on Defect Size, Type and Anatomical Location

CDH is the most severe diaphragm defects compared to other, less frequent defects such as incomplete muscularization of the diaphragm (diaphragmatic eventration) or the presence of just a thin layer of non-muscular tissue (sac hernia). Subtypes are identified by the size and anatomical location of the herniation. Most prevalent are Bochdalek hernias, which are mostly left-sided (20). Prenatal predictors for survival include associated malformations (21), defect size (7), lung volume (22), liver herniation (23), stomach position (24, 25), and lung-to-head ratio (26, 27). Other predictors include birth weight, Apgar score, respiratory parameters, cardiac anomalies, chromosomal changes, and pulmonary hypertension (28–30).

## The Relation of Defect Size and Genetic Alterations

Larger diaphragms defects are associated with a higher mortality rate, the prevalence of associated anatomical malformations as well as the number of associated anatomical malformations (21). We hypothesized that large continuous locus or gene changes (e.g., 15q26 loss, 17q12 loss; see Table 1) can modify multiple genes involved in diaphragm formation, and impact the development of the embryo in general. In contrast, small deletions or Single Nucleotide Variants (SNVs) as seen in for instance *FBN1, TGFB3*, and *SLC2A10* (see Table 2) will be associated with smaller defects. Therefore, we evaluated whether the size of the defect was associated with the finding of “a pathogenic genomic variant” and/or “a genetic syndrome.” We compared the genetic test results and the defect size classification (*n* = 336). Statistical analysis did not indicate associations of the defect size with an different, uncommon genetic test result. What we did observed was that patients with no or little follow-up revealed associations (*P* < 0.001). In this category patients are present lacking a registered defect size or registered genetic test. This category includes patients who have not been subjected to an intervention due to intrauterine fetal demise or termination of pregnancy. In the Netherlands, pregnancies in which severe genetic anomalies (e.g., Edwards syndrome, Patau syndrome) or structural malformations are observed that are incompatible with life, are often terminated. The CDH defect size is not determined in those cases (see Table 1). Therefore, a complete genetic and phenotypic evaluation and subsequent association analysis in this particular group is difficult and often not performed.

**Table 1:** Pathogenic alterations in CDH patients of which the defect size was not registered.

**Defect size (*n*)**	**Syndrome (*n*)**	***n***	**Death**	**Chromosome**	**Type**	**Inheritance**	**Zygosity**	**Genetic change**
NR (*n* = 41)	Microdeletion	1	NR	3p26.3-p25.3	Loss	*de novo*	het	arr[hg18] 3p26.3-p25.3 (0–9398383) x1
	Microduplication		NR	11q23.3-q25	Gain	*de novo*	het	arr[hg18] 11q23.3-q25 (16192532–134452384) x3
	Microdeletion	1	NR	5p15	Loss	*de novo*	het	arr[hg19] 5p15 (0–37,299,510) x1,
	Microduplication		NR	12p13.3	Gain	*de novo*	het	arr[hg19] 12p13.31 (9,909,002–10,021,222) x 3
	Cornelia de lange	1	N	5p13.2	Missense	*de novo*	het	NM_1334333 (*NIPBL*): c.3574G>A; p. (Glu1192Lys)
	Microduplication	1	T	7q11.23	Gain	*de novo*	het	arr[hg18] 7q11.23 (72,701,018–74,143,000)
	Microduplication	1	NR	8p23	Gain	*ut*	het	46, XY, der (8) t (3;8) (p23; p23.1)
	Microduplication	1	D	9p24.3-p13.1	Gain	*de novo*	het	arr[hg18] 9p24.3p13.1 (0–39,155,853) x4, arr[hg18]9p13.1p11.2 (39,155,853–46,468,856) x3
	Microdeletion	1	T	9q31.1q31.2	Loss	*de novo*	het	arr[hg19] 9q31.1q31.2 (105,034,238–111,044,933) x1
	Trisomy 9	1	I	9	Aneuploidy	*de novo*	het	47, XX, +9(20)/46, XX (4)
	Mosaic MYRF gene	1	N	11q12.2	Splicing	*de novo*	het	NM_001127392.2 (*MYRF*): c.46+2T>C(r.spl?)
	Pallister Killian syndrome	3	T (1), NR (2)	12p10	Gain	*de novo*	het	47, XX/XY, +i (12) (p10)
	Microduplication	1	D	12q24.3	Gain	*ut*	het	46, XY, der (12) t (11,12) (q23.3; q24.3)
	Microdeletion	1	T	13q12	Loss	*de novo*	het	46, XY, del (13) (q12?) (8)/46, XY (35)
	Microdeletion	1	T	13q21.31q32.3	Loss	*de novo*	het	arr[hg19]13q21.31q32.3 (64,535,372–98,354,979) x1
	Patau syndrome	3	T (1), D (1), NR (1)	13	Aneuploidy	*de novo*	het	47, XX +13
	Isochromosome 14q	1	N	14q10	Gain	*de novo*	het	46, XX, i (14) (q10) (3)/46, XX (22)
	Microduplication	1	NR	15		*ut*	het	46, XX, der (15) t (2;15)
	Microdeletion	1	D	15q26	Loss	*de novo*	het	46, XY, t (1;14) (p22; q13), inv (6) (p25q22), del (15) (q26)
	Edward's syndrome	16	T (3), I (1), N (2), D (3), NR (9)	18	Aneuploidy	*de novo*	het	47 XX / XY + 18
	Down syndrome	1	NR	21	Aneuploidy	*de novo*	het	47, XX +21
	Cat eye syndrome	1	T	22q11.1q11.21	Gain	*de novo*	het	arr [hg19] 22q11.1q11.21 (14,449,498–17,017,139) x4
	XY reversal*	2	D (2)	XY	?	*de novo*	het	?

*Genetic tests included karyotyping, SNP array or Whole exome sequencing. AR, Autosomal recessive; XLR, X-linked recessive; CH, compound heterozygote; n, number of patients; ut, unbalanced translocation*.

**Table 2:** Pathogenic alterations in CDH patients of which the defect size was registered.

**Defect size (*n*)**	**Syndrome (*n*)**	***n***	**Death**	**Chromosome**	**Type**	**Inheritance**	**Zygosity**	**Genetic change**
A (*n* = 10)	Wolf Hirschshorn Syndrome	1	NR	4p156.3	Loss	*de novo*	het	46, XY FISH: ish del (4) (p16.3p16.3) (D4S96-)
	Louys-Dietz syndrome V	1	NR	14q24	Frameshift	*AD*	het	NM_003239.4 (*TGFB3*): c.232del.G, p. (Glu78fs)
	Marfan syndrome	1	NR	15q21.1	Frameshift	*AD*	het	NM_000138.5 (*FBN1*):c1301_1302del, p. (Tyr434Serfs*17)
	Microdeletion	1	NR	16p13.3	Loss	*de novo*	het	46, XY arr[hg18] 16p13.3 (154,014–174,381) x1
	Arterial tortuosity syndrome	1	NR	20q13	Missense	*AR*	hom	NM_030777.4 (*SLC2A10*): c.127 6G>T, p. (Gly426Trp)
	Down syndrome	4	D (1), NR (3)	21	Aneuploidy	*de novo*	het	47, XX / 47, XY + 21
	Down syndrome	1	NR	21	Aneuploidy	*ut*	het	46, XY, t (15;21) (p12; p12)
B (*n* = 4)	Microduplication	1	NR	4p15.2p14	Gain	*de novo*	het	arr [hg18] 4p15.2p14 (224,500,018–38,700,366) x3
	Sotos syndrome	1	NR	5q35.2	Missense	*de novo*	het	NM_022455.5 (*NSD1*): c.5685C>G, p. (Cys1895Tyrp)
	Microduplication	1	NR	7q31.33–36.3	Gain	*de novo*	het	arr[hg19]7q31.33q36.3 (125839750_159124173) x3[0.2]/arr[hg19]7q31.33q36.3 (125839750_159124173) x4[0.1]
	Microdeletion	1	D	8p23.1	Loss	*de novo*	het	arr[hg18] 8p23.1 (8,139,051–12,619,015) x1
C (*n* = 5)	Fraser syndrome	1	NR	9p22.3	Splicing	*de novo*	het	NM_144966.7 (*FREM1*): c.5334 + 1G > A (r.spl?)
	Microdeletion			9p22.3	Loss	*Inherited*	het	arr[hg18] 9p22.3 (14,871,409–14,938,830) x1
	Prader Willi	1	NR	15q11	Gain	*de novo*	het	arr[hg18]15q11.2q13.1 (20,319,702–26,143,385) x3
	Microdeletion	1	NR	17q12	Loss	*de novo*	het	arr[hg19] 17q12 (34815551_36249430) x1
	Congenital disorder of glycosylation	1	NR	Xp11.23	Loss	*de novo*	het	NM_001042498 (*SLC35A2*): c.753delG, p.(Trp251fs)
	XY reversal	1	D	XY	?	*de novo*	?	–*
D (*n* = 2)	Microdeletion	1	N	15q26	Loss	*de novo*	het	arr[hg18] chr15:80,689,404–82,938,351 x 1 and
				17p12			het	arr[hg18] chr17:14049619–15497020 x1
	Microdeletion	1	D	22q11.2	Gain	*ut*	het	47, XY, +der (22) t (11;22) (q23.3; q11.2) mat

*Genetic tests included karyotyping, SNP array or Whole exome sequencing. AR, Autosomal recessive; XLR, X-linked recessive; CH, compound heterozygote; n, number of patients; ut, unbalanced translocation*.

## Isolated CDH and Complex CDH

CDH may present as an isolated anomaly (isolated-CDH) or patients can have one or more additional anomalies (CDH-complex) (1, 31). Anomalies can be found in all body sites; cardiac anomalies, anomalies of the urogenital system, limb malformations, nervous system anomalies, orofacial clefts, and gastrointestinal anomalies including intestinal atresia (3, 32). Zaiss et al. described syndromic clinical features such as hypertelorism not assigned to a specific syndrome in 7.7% of studied patients (32). Pathogenic genetic alterations—both in complex and in isolated CDH—are associated with a worse prognosis (33). Moreover, *de novo* pathogenic alterations are seen more often in complex CDH (34–36). Phenotypical complex patients could be more likely to receive a genetic test. In our cohort, genetic test results were described for patients with associated anomalies (*n* = 207) and for patients without associated anomalies (*n* = 311). Thus, there was not a priory bias in this respect (*p* = 0.923). Twenty patients with associated anomalies had pathogenic genetic alterations vs. one with isolated CDH (*P* < 0.001). Main outcome parameters of the Erasmus MC-Sophia Children's Hospital, Rotterdam, the Netherlands CDH cohort are depicted in Tables 3, 4. Full cohort descriptions and analysis methods are described in Supplementary Tables S1, S2.

**Table 3:** Cohort description of output measures and genetic evaluation.

**Group**	**Characteristic**	**Genetic test (*n* = 530)**	**No genetic test (*n* = 275)**	**Total (*n*)**	***P***	**Abnormal genetic test (*n* = 62)**	**No genetic test (*n* = 275)**	**No pathogenic changes (*n* = 468)**	**Total (*n*)**	***P***
Sex	F	238^a^ (44.9%)	120^a^ (43.6%)	358 (44.5%)	0.824	34^a^ (54.8%)	120^a^ (43.6%)	204^a^ (43.6%)	358 (44.5%)	0.502
	M	285^a^ (53.8%)	150^a^ (54.5%)	435 (54.0%)		27^a^ (43.5%)	150^a^ (54.5%)	258^a^ (55.1%)	435 (54.0%)	
	O	7^a^ (1.3%)	5^a^ (1.8%)	12 (1.5%)		1^a^ (1.6%)	5^a^ (1.8%)	6^a^ (1.3%)	12 (1.5%)	
Associated anomalies	CDH-C	207^a^ (39.1%)	104^a^ (37.8%)	311 (38.6%)	0.923	56^a^ (90.3%)	104^b^ (37.8%)	151^b^ (32.3%)	311 (38.6%)	4.5658E-16
	CDH-I	311^a^ (58.7%)	164^a^ (59.6%)	475 (59.0%)		6^a^ (9.7%)	164^b^ (59.6%)	305^b^ (65.2%)	475 (59.0%)	
	CDH-MD	12^a^ (2.3%)	7^a^ (2.5%)	19 (2.4%)		0^a^ (0.0%)	7^a^ (2.5%)	12^a^ (2.6%)	19 (2.4%)	
Location of defect	Bilateral	4^a^ (0.8%)	6^a^ (2.2%)	10 (1.2%)	0.005998	0^a^ (0.0%)	6^a^ (2.2%)	4^a^ (0.9%)	10 (1.2%)	0.004092
	Eventration	17^a^ (3.2%)	1^b^ (0.4%)	18 (2.2%)		1^a, b^ (1.6%)	1^b^ (0.4%)	16^a^ (3.4%)	18 (2.2%)	
	Left	415^a^ (78.3%)	199^a^ (72.4%)	614 (76.3%)		48^a^ (77.4%)	199^a^ (72.4%)	367^a^ (78.4%)	614 (76.3%)	
	POE	4^a^ (0.8%)	2^a^ (0.7%)	6 (0.7%)		2^a^ (3.2%)	2^a^ (0.7%)	2^a^ (0.4%)	6 (0.7%)	
	Right	73^a^ (13.8%)	58^b^ (21.1%)	131 (16.3%)		7^a, b^ (11.3%)	58^b^ (21.1%)	66^a^ (14.1%)	131 (16.3%)	
	MD	17^a^ (3.2%)	9^a^ (3.3%)	26 (3.2%)		4^a^ (6.5%)	9^a^ (3.3%)	13^a^ (2.8%)	26 (3.2%)	
Defect size	A	97^a^ (18.3%)	19^b^ (6.9%)	116 (14.4%)	1.3023E-41	10^a, b^ (16.1%)	19^b^ (6.9%)	87^a^ (18.6%)	116 (14.4%)	1.3224E-44
	B	50^a^ (9.4%)	2^b^ (0.7%)	52 (6.5%)		4^a^ (6.5%)	2^b^ (0.7%)	46^a^ (9.8%)	52 (6.5%)	
	C	157^a^ (29.6%)	12^b^ (4.4%)	169 (21.0%)		5^a^ (8.1%)	12^a^ (4.4%)	152^b^ (32.5%)	169 (21.0%)	
	D	32^a^ (6.0%)	0^b^ (0.0%)	32 (4.0%)		2^a^ (3.2%)	0^b^ (0.0%)	30^a^ (6.4%)	32 (4.0%)	
	NR	194^a^ (36.6%)	242^b^ (88.0%)	436 (54.2%)		41^a^ (66.1%)	242^b^ (88.0%)	153^c^ (32.7%)	436 (54.2%)	
Timing of test	MD-genetic test	–	–	–	–	13^a^ (21.0%)	0^b^ (21.0%)	88^a^ (18.8.0%)	101 (12.5%)	8.4554E-167
	MD-no genetic test	–	–	–		0^a^ (0.0%)	127^b^ (46.2%)	0^a^ (0.0%)	127 (15.8%)	
	Postnatal-genetic test	–	–	–		16^a^ (25.8%)	0^b^ (0%)	101^a^ (21.6%)	117 (14.5%)	
	Postnatal-no genetic test	–	–	–		0^a^ (0.0%)	96^b^ (34.9%)	0^a^ (0.0%)	96 (11.9%)	
	Prenatal-genetic test	–	–	–		33^a^ (53.2%)	0^b^ (0%)	279^a^ (59.6%)	312 (38.8%)	
	Prenatal-no genetic test	–	–	–		0^a^ (0.0%)	52^b^ (18.9%)	0^a^ (0.0%)	52 (6.5%)	

*In total, 530 out of 805 patients received a genetic test. Defect size (A–D) was described in 369 patients. Defect sizes are classified from A to D as described in the method section. A is the smallest defect size and D a (near) absence of the diaphragm. Within a column each characteristic that does not share a subscript letter (^a−b^) differs significantly from those with different subscript letters (^a−b^) whose column proportions do not differ significantly from each other at the 0.05 level. For instance, more patients with associated anomalies have an abnormal test and vice versa more patients with an isolated defect have no abnormal test (P < 0.001). Patients with defect size A stand apart from the other defect sizes in respect to the number of abnormal genetic tests, C in having no genetic test and having no pathogenic alteration (P < 0.001). There are differences in having no genetic test, having an abnormal test result and having a normal test result comparing post- and pre-natal subgroups (P < 0.001). Trisomy 13, 18, and 21 were evaluated in 530 patients and more than half of the patients received at least karyotyping or SNP-array. A full cohort description is available in Supplementary Table S1. Complete statistical comparison of patients with a genetic test is depicted in Supplementary Table S2. MD, Missing data; CDH-C, CDH patients with associated defects; CDH-I, CDH patients without other associated defects; CDH-MD, CDH patients in which no additional information was registered; POE, Paraoesophageal hernia; EV, Eventration; BL, Bilateral hernia; AGT, abnormal genetic test; NPC, no pathogenic changes*.

**Table 4:** Significant differences in output measures of patients with a genetic test.

**Group**	**Characteristic**	**Abnormal genetic test (*n* = 62)**	**No pathogenic changes (*n* = 468)**	***P***
Associated anomalies	CDH-complex (*n* = 207)	56^a^ (27.1%)	151^a^ (72.9%)	1,432E-14
	CDH-isolated (*n* = 311)	6^b^ (1.9%)	305^b^ (98.1%)	
	CDH-unknown (*n* = 12)	0^a,b^ (0.0%)	12^a,b^ (100.0%)	
Defect size	A (*n* = 97)	10^a,b^ (10.3%)	87^a,b^ (89.7%)	0.000006
	B (*n* = 50)	4^a,b^ (8.0%)	46^a,b^ (92.0%)	
	C (*n* = 157)	5b (3.2%)	152^b^ (96.8%)	
	D (*n* = 32)	2^a,b^ (6.3%)	30^a,b^ (93.8%)	
	NR (*n* = 194)	41^a^ (21.1%)	153^a^ (78.9%)	
Type of genetic test	Karyotyping	297 (56.0%)		
	WES	51 (9.6%)		
	Array	362 (68.3%)		
	Trisomy 13, 18, 21*	530 (100%)		

*Significant differences when evaluating only patients with a genetic test. Trisomy 13, 18, and 21 were evaluated in 530 patients and more than half of the patients received at least karyotyping or SNP-array. An abnormal genetic test is seen more often in complex-CDH (P < 0.001) and defect size C differs from the missing data category (P < 0.001) as substantially more abnormal genetic tests are described in the later. Within a column each characteristic measure that does not share a subscript letter (^a−b^) differs significantly from those with different subscript letters (^a−b^) whose column proportions do not differ significantly from each other at the 0.05 level. WES, whole exome sequencing; MD, Missing data; CDH-C, CDH patients with associated defects; CDH-I, CDH patients without other associated defects; CDH-MD, CDH patients in which no additional information was registered; POE, Paraoesophageal hernia; EV, Eventration; BL, Bilateral hernia; AGT, abnormal genetic test; NPC, no pathogenic changes*.

Comparing features of isolated CDH and complex CDH is difficult, depending on how accurately these two groups can be distinguished. Not all patients receive the same phenotypical evaluation and registration is sometimes incomplete. For instance, not all associated anatomical malformations are detectable with ultrasound. Nevertheless, increased resolution of prenatal ultrasound over time has improved the detection of associated anatomical malformations. Neurological symptoms could develop at later age and are not noticeable during the first months or years of development. Furthermore, not all symptoms observed during often organ specific evaluations of medical subspecialities. For instance, postnatal monitoring is essential to detect any associated neurological or ophthalmological symptoms. CDH registries would benefit from regular re-evaluation of these outcome measures. In short, there is a level of uncertainty in registries regarding which patients have no associated anomalies, have no associated anomalies detected, or have no associated anomalies registered.

## Genetic Associations and Co-morbidity

Long-term complications in children born with CDH include chronic lung disease, feeding difficulties, gastro-esophageal reflux, growth failure, scoliosis, chest asymmetry, neurodevelopmental delay, and sensorineural hearing loss (37, 38). These co-morbidities can be either a direct or indirect consequence of the CDH or be a consequence of the treatment. Damaging *de novo* variations in both isolated CDH and complex CDH-complex have been found associated with pulmonary hypertension, higher mortality rate, and worse neurodevelopmental outcome (33). There is a large difference in survival rates between patients with or without persistent pulmonary hypertension (39) and bronchopulmonary sequestration (40). The genetic contribution to bronchopulmonary sequestration etiology is unknown. Mutations in *BMPR2* (41, 42) and several *SMAD* signaling molecule genes have been associated with the development of pulmonary hypertension in adults and children (43–45). A striking association between TGF-β/SMAD signaling and pulmonary hypertension has been reported in CDH, as the CDH lungs had increased miR-200b expression and decreased TGF-β/SMAD signaling (46). Increasing miR-200b decreases the TGF-β signaling and reduces lung hypoplasia in a nitrofen induced congenital diaphragmatic hernia -pulmonary hypertension rat model (46). Similarly, Pereira-Terra and colleagues described a specific micro-RNA signature in tracheal aspirate fluid, upregulation of miR-200b and miR-10a and decreased TGFB signaling (47). Patients with mutations in genes from this pathway have connective tissue disorders (48). In patients and mice, several genetic factors have been associated to lung and cardiac abnormalities (2, 49–52). CDH has been found in patients with connective tissue disorders such as Marfan syndrome (53), Loeys-Dietz Syndrome (54, 55) and arterial tortuosity syndrome (56). Patients with these connective tissue disorders are at increased risk of cardiovascular problems (57, 58) later in life. Abnormal retinoic acid signaling can result in a diaphragm defect (59). Patients with variants in *STRA6* and *RARB*-receptors and deletions of *RBP1* at chromosome 3q22 (60, 61) in the retinoic acid signaling pathway have ophthalmic symptoms (62, 63). Patients with CDH may have other eye defects as well (64, 65). These occurrences of direct genotype-phenotype correlations stress the importance of genetic diagnostic screening to inform parents and patients about possible co-morbidities.

## CDH Is a Complex Genetic Disorder

CDH is a multifactorial disease but neither environmental nor genetic contributions have been fully characterized. Maternal morbidities during pregnancy such as pre-gestational hypertension (66) and pre-existent maternal obesity (67–69) are associated with an increased risk for development of CDH in the fetus. Several other environmental factors have been associated with an increased risk: antidepressant medication (70), antibacterial medication (71), exposure to fungicides (72), the immunosuppressant drug mycophenolate mofetil (73), methotrexate use (74), exposure to cadmium (75), pesticides (76), hairspray use (77), alcohol intake (69, 77–79), and smoking (75, 78, 80). However, to what extent these associations impact diaphragm development and the onset of CDH is not known. The mother's nutrient intake during pregnancy is associated as well (81, 82); reduced vitamin A intake during pregnancy has the strongest associations with CDH (83, 84). Vitamin A shortage can be detected postnatally (85). It is hard to determine whether environmental factors explain some of the non-genetic contributions on a population level or to what extent the environment interacts with the processes disturbed by genetic anomalies. Epigenetic differences acquired during the life span can be detected between monozygotic twin pairs (86–88). Evaluating these differences—and the resulting gene expression changes—is an interesting approach. There are methods to overcome cellular heterogeneity and if epigenetic changes are present in blood these can be compared between patient and sibling (89–91).

The exact heritability—the contribution of genetic factors—is difficult to determine, in light of the relatively low disease incidence, the high mortality limiting vertical transmission and the limited numbers of twin pregnancies (92, 93). Heritability can be estimated using twin studies. For CDH, the concordance rates in dizygotic and monozygotic twins are comparable. Fifty-three monozygotic twins have been described, of whom 12 were concordant for CDH (2, 92). In our cohort, 24 twin pairs (15 dizygotic, 8 monozygotic, and one same sex twin pair of whom no genetic material was available to determine zygosity) are described. One dizygotic and one monozygotic twin pair were concordant for CDH. To reduce the effect of technical noise in twin comparisons, we used different alignment techniques, variant callers and statistics (see Supplementary Table S3). Neither the larger CNVs (94) nor SNVs (see Supplementary Table S3) differed between these twin siblings. Differences in phenotype can also be the result of twin-to-twin perfusion differences. Furthermore, single nucleotide changes could be located outside the coding sequence or at very low frequency, and then could not be detected with exome sequencing.

Somatic mosaicism is difficult to determine when the affected tissue or cells are missing. The mutated diaphragmatic cells might not have survived in sufficient quantities and, therefore, be undetectable with sequencing technologies (95). In line with this, whole genome sequencing did not find causative somatic variants in diaphragm biopsies (96, 97). In contrast, germline *de novo* variants are often present (33, 96–98). Females have a higher burden of *de novo* variants (98), suggesting a female protective model. Large cohort descriptions about sibling recurrence rate (92) or familial CDH are not available. In our cohort, only a few familial cases are known (<1%). Still, CDH is described to segregate through families (1) and/or present as a monogenetic disorder following autosomal dominant (53, 98–109), autosomal recessive (62, 110), or X-linked (111–113) inheritance patterns.

Depending on the specific family the monogenetic disorder has CDH is either a common or a less prevalent feature. More than 100 (candidate) genes have been described, mostly identified from animal models or monogenetic syndromes (2, 19). Monogenetic syndromes often have distinct phenotypical features and have been reviewed by Longoni et al. and Yu et al. (20, 114). Monogenetic syndromes in which CDH is a frequent feature are, for instance, autosomal recessive Donnai Barrow syndrome (OMIM: #22248, *LRP2* gene), syndromic microphthalmia (#601186, #615524, *STRA6, RARB*), and autosomal dominant cardiac-urogenital syndrome (#3618280, *MYRF* gene). Associated phenotypes in these syndromes are congenital heart defects, sensorineural hearing loss, microphthalmia, genitourinary malformations, craniosynostosis and myopia with each of these syndromes its distinct features. Detailed phenotyping might be crucial in diagnosing clusters of CDH patients: either “phenotype first” and searching for an overlapping gene or “genotype first” and searching if patients with the same affected gene have an overlapping phenotype. Interestingly, Fryns syndrome and also Pentalogy of Cantrell have CDH as a defining feature; yet the gene or genes responsible for these conditions are not yet known.

CNV studies reported overlapping deletions and duplications, such as duplications of 11q23-qter (115), 16p11.2p duplications (15, 18), 17q12 deletions (15, 18, 116, 117), and 5p15.2 deletions (15). By prioritizing and sequencing the genes within these CNVs in other patients, new disease genes have been discovered. For example, in the 8p23.1 deletions (118–120), *GATA4* (50) and *SOX7* (121) and in case of 8q23.1 deletions (18), *ZFPM2* (122) are the genes likely contributing to CDH. 15q26 deletions (120, 123) and subsequent sequencing implicate *NR2F2* as a disease gene (124). For 1q41–1q42 deletions, one duplication disrupting the *HLX* gene and subsequent *HLX* gene variants have been described (15, 18, 125–128). Constraint coding regions are enriched for *de novo* variants (104), and using variant evaluation guidelines of rare *de novo* changes in these types of constraint genes (129) result in a likely pathogenic or pathogenic classification, especially if variants result in reduced amounts of protein.

**Figure 1:**
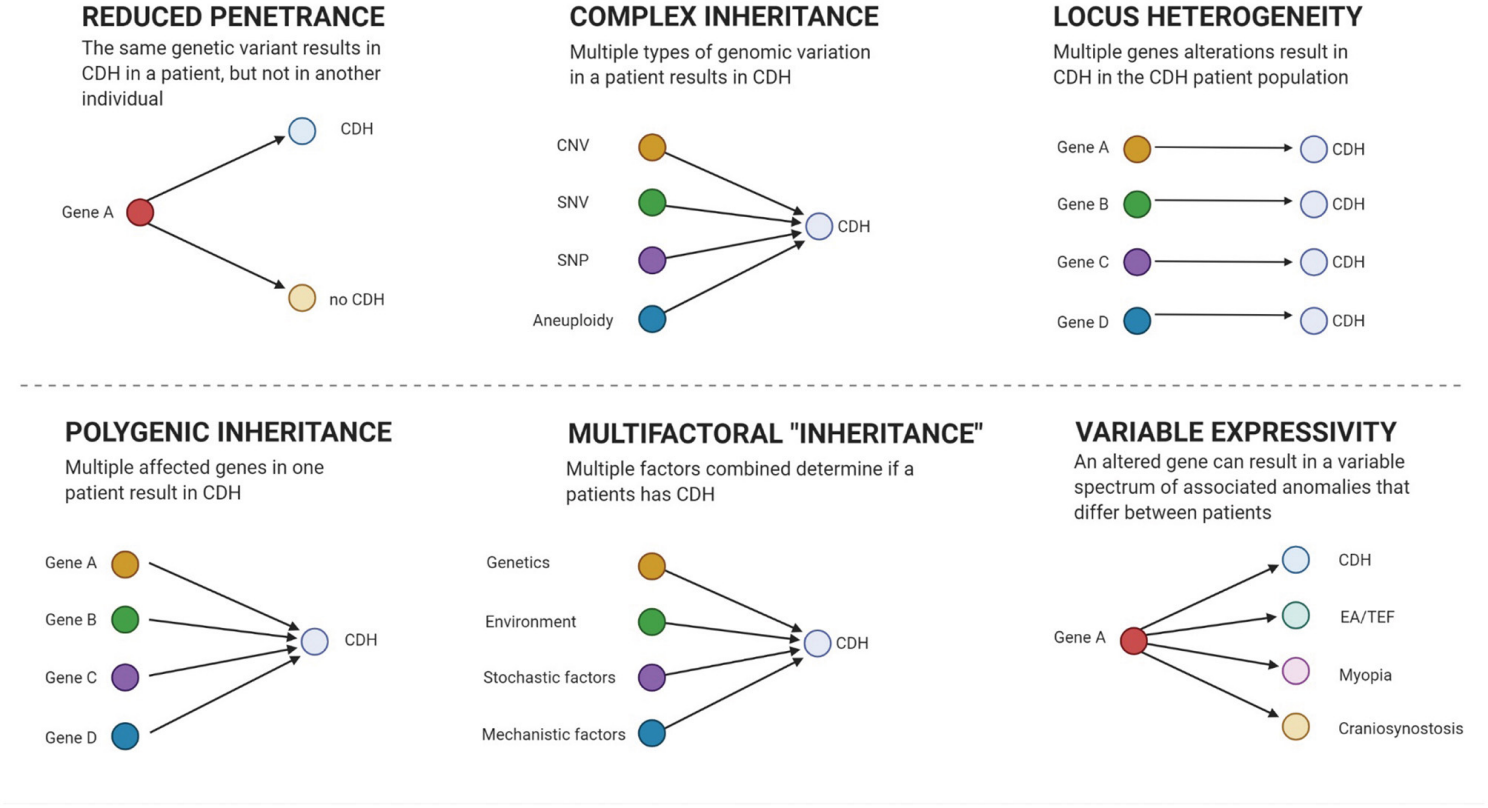
Genetic models. Figure created with BioRender.com.

Interpretation of genetic results can be hindered by reduced penetrance (18, 122) and variable expressivity (2) that may mask the causal culprit in segregation analysis (see Figure 1). Polygenic inheritance (51), locus heterogeneity (33, 34, 130), and contributions of different kinds of genetic variation (17, 114) mask culprits from innocent bystanders. Therefore, large patient and control samples sizes are required to have enough power to classify variants into “benign,” “causal,” or “contributing.”

## From Pathogenic Alteration to CDH

Finding a genetic variant predicted to be deleterious is only the first step in proving the functional effect of this DNA alteration. This is especially true for missense changes, in-frame insertion-deletions and copy number variations. Often there is only *in-silico* evidence regarding the impact of a variant on gene function and the way in which the disturbed gene function affects a biological pathway or mechanism. What is lacking is proof how a specific deleterious variant lead to defective diaphragm formation. Unfortunately, for most likely pathogenic CNVs and SNVs, the assumed functional consequence is based on the genetic alteration itself: i.e., copy number loss or nonsense variant is assumed to result in reduced amounts of mRNA expression and protein. Deleterious *de novo* missense variants and in-frame insertion-deletions in conserved coding regions are more difficult to relate to a likely functional consequence and is often on *in-silico* surveys. Improving the *in-vitro* evaluation of candidate variants is crucial in distinguishing causal variants from non-causal variants. These experiments require tremendous effort and can be complicated by the presence of more than one candidate alteration.

Detecting a deleterious variant in a gene in multiple patients helps prioritizing candidate genes for function evaluation and studies using animal models. In a large cohort (*n* = 827), seven syndromic and four recurrent CNVs were identified (104). Some of these have already been associated with CDH; e.g., 17q12 deletions, 16p13.1 duplications, 22q11 deletions, and 21q22 duplications. Furthermore, 87 CNVs were *de novo*, of which 54 were large (>2 Mb) deletions (104). Although non-recurrent, at least a proportion of these large *de novo* deletions are likely to be related to the patient's phenotype. Ten genes were enriched for *de novo* variants, of which mitochondrial lon peptidase 1 (*LONP1*) and Aly/REF export factor (*ALYREF*) were the most promising candidate disease genes. *LONP1, MYRF* as well as *ZFPM2* reached or approached genome wide significance when a variant burden test was performed for all deleterious changes (i.e., including inherited variants) (104). Combining multiple “omics” and *in-vitro* translational approaches can potentially bridge the gap between genetic findings and animal models.

In animal models, fewer progenitors reaching the PPF at the proper developmental due to decreased proliferation, increased apoptosis, migration defects or failure to differentiate in their proper cell fates have been proposed as causes for CDH (131–134). Disturbances in specific processes such as retinoic acid signaling or muscle connective tissue formation were initially discovered in animal experiments; genes associated with these pathways or processes were subsequently found altered in patients (132, 135–138). Additionally, disturbed processes can be identified using gene enrichment strategies to find common denominators in the affected genes and loci. Longoni and colleagues described the enrichment of rare, likely deleterious variants in CDH patients of genes derived from mouse PPF embryonic transcriptomes (139), known human disease genes, their protein interaction partners and candidate genes from CNV hotspots (35). Often, these alterations were inherited and implicate non-Mendelian inheritance patterns. On the individual level, these changes can be regarded as risk factors. Combined, these changes may affect a biological pathway to such an extent that they result in CDH. Assigning such a pathway or process—for instance how these gene variants disturb myoblast progenitor cell proliferation or migration—is not easy. Animal models are not perfect, although they provide evidence of involvement of a gene when it is knocked-out and in which cases the animals develop CDH at a certain frequency. However, this procedure hardly ever takes into account that genetic variation is mostly not a complete loss-of-function of a gene. Missense variants, copy number gains and heterozygous changes could—and likely do—differ in impact or mechanism of action. Thus, in these cases, knock-out models either over- or underestimate the effect of a genetic variant.

In some cases, specific variants can be associated with the causative mechanism; e.g., the association of *FBN1* variants in Marfan syndrome (53) and defects in the connective tissue. Indeed, our cohort included patients with *FBN1* and *TGFB3* alterations. In other patients, the affected pathway is known; e.g., patients with deletions of *NR2F2* (123) have a defect in a gene that codes for a receptor that is activated by retinoic acid signaling (140). Of other genes, we know that they interact with other disease genes, are expressed in the developing diaphragm and are also associated with retinoic acid signaling (e.g., *ZFPM2, GATA4*). A small difference in spatial and temporal binding and organ-specific combination of transcription factors have been suggested as links between the different syndromes with CDH (141). Most of the deleterious CNVs and aneuploidies are assumed pathogenic and the most likely cause of the diaphragm defect. However, how these—often continuous gene deletions—in patients impact diaphragm formation and subsequently result in CDH remains unclear.

## Temporal Screening Bias

Technologies have a different resolution to detect genomic changes ranging from chromosome arms, several mega-bases to single nucleotide level. Initially, patients were evaluated with karyotyping, MLPA and QF-PCR, with which only aneuploidies or chromosome (band) level changes could be detected. At the Erasmus MC-Sophia Children's Hospital, SNP-array was introduced in 2010 and is standard practice in case of ultrasound abnormalities since 2012. The use of SNP arrays increased the detection resolution to gains and losses of several from mb to kilobases. Many patients in our cohort have retrospectively been re-evaluated with SNP-array. In 10.9% of patients a pathogenic change was. Similarly, 10.4% of patients registered in the EUROCAT registry (1980–2009) have a chromosomal anomaly, genetic syndrome or microdeletion (3). This was before the NGS era, and the findings mostly represent the larger genetic changes with a large phenotypic effect. Whole exome sequencing was introduced in our clinic more recently (2015), and initially only used to evaluate the more complex patients. Restoring the temporal screening bias by screening large historical cohorts of patients and subsequent evaluating potential associations between genetic factors and long-term morbidity can benefit the future and today's patients and parents.

## Collaboration Is Key

Combining disease cohorts revealed that damaging *de novo* alterations are associated with the more severe and complex phenotypes (33, 130). This strategy was pivotal in identifying disease genes (98, 104, 130, 142). The success of this effort stresses the importance of collaborations such as the DHREAMS consortium (http://www.cdhgenetics.com). Trio whole genome-based approaches are recommended, as these enable to simultaneously determine different types of genetic variation. Additionally, this technique is suited for continuous re-analysis. By combining and sequencing these cohorts, the CDH-EURO consortium (143) and Congenital Diaphragmatic Hernia Study Group (144) can add to endeavors of the DHREAMS consortium. This will enable to identify genes that are more often affected in patients than by chance alone, and will allow manageable numbers of required functional tests and animal models. For collaborations to work, samples need to be stored in well-managed biobanks and data should be meticulously archived for later re-analysis or re-evaluation. New challenges for these biobanks and data archiving and sharing are privacy regulations (145). Sharing of patient material and data should consider the privacy of participants and their families but also acknowledge the efforts of stakeholders such as researchers and clinicians (146). An ethical and legal balance should be sought weighing the privacy needs of individual patients against the medical benefits of the patient population.

## Conclusions

Diagnostic yields of up to 37% using next generation sequencing have been proposed. These yields are reached when, in addition to genes from known monogenetic syndromes, heterozygous *de novo* variants in genes expressed at the proper time-point in relevant tissue in animal models are classified as likely pathogenic (105). Importantly, heritability and diagnostic yield are calculated on a population level. From a patient's or parents' perspective it matters the most to know (1) if they themselves or ***their*** children **have** *or* **do not have** genetic changes in their genome explaining the CDH, (2) if subsequent children or patients' offspring are at risk of CDH, and (3) what the consequences of these changes are for the prognosis and/or the probability of complications. CDH is now mostly detected prenatally; consequently, fast, accurate, and predictive genetic diagnostics are increasingly needed. As about a third of patients have a *de novo* variant in the coding region (104). For parents to make informed choices, it is vital to knowing if a genetic variant detected in their child is causal or benign, and what the predicted consequences are of this variant.

## Author Contributions

EB, AdK, and DT: conceptualization and funding acquisition. RB and WvI: methodology and software. EB: validation, visualization, and project administration. KvW, SO, EB, and RB: formal analysis. EB and RB: investigation. AE, DT, and RW: resources. EB, RB, NP, and DV: data curation. EB and KvW: writing—original draft preparation. NP, AdK, MvD, SO, RW, AE, DT, RR, CB, HR, DV, WvI, FS, HB, RB, and JS: writing—review and editing. AdK and EB: supervision. All authors have read and agreed to the published version of the manuscript.

## Funding

This work was supported by the Sophia Foundation for Scientific Research [Grant Numbers 493 (DT and AdK) and S13-09 (DT, AdK, and EB)].

## Conflict of Interest

The authors declare that the research was conducted in the absence of any commercial or financial relationships that could be construed as a potential conflict of interest.

## Publisher's Note

All claims expressed in this article are solely those of the authors and do not necessarily represent those of their affiliated organizations, or those of the publisher, the editors and the reviewers. Any product that may be evaluated in this article, or claim that may be made by its manufacturer, is not guaranteed or endorsed by the publisher.
