# Supplementary material for: Unraveling the Genetics of Congenital Diaphragmatic Hernia: An Ongoing Challenge
Source: Front Pediatr. 2022 Feb 3;9:800915. doi: 10.3389/fped.2021.800915 (PMC8852845; doi:10.3389/fped.2021.800915)
Supplement: Supplementary file 1 [file Data_Sheet_1.pdf]

S1 Cohort description and statistics

Methods

Patients and setting

We retrospectively evaluated data of all registered patients diagnosed with CDH admitted and/or referred to the Erasmus MC- Sophia Children's Hospital from 1972 till September 2020 (n=805). This hospital serves as a level 4 referral center for other academic hospitals for CDH diagnosis and treatment. Research involving human participants was performed in routine diagnostic procedures or included in a protocol approved by the Erasmus University Medical Centre’s ethics review board (MEC no.193.948/2000/159, addendum Nos. 1 and 2 and MEC-2021-0064.). Parental informed consent included genetic studies in both siblings of discordant monozygotic twins and their parents. CDH had been detected either prenatally through ultrasound screening or postnatally. Genetic diagnosis was retrieved from the medical records (i.e., the results of karyotyping, (targeted) gene sequencing, non-invasive prenatal testing, quantitative PCR and/or SNP array) and combined with clinical and follow-up data in an interactive database.

Analysis of defect size

The Boston Classification classifies defect size from A to D, using a scheme in which A represents a defect entirely surrounded by muscle, and D the largest defect size in which the diaphragm is fully or nearly absent [1; 2]. Unfortunately, the Boston Classification was not always used to describe the size of the defect. To allow a statistical comparison between registered defect size and a genetic test result, we increased the number of registered defect sizes by classifying primarily repaired defects under A (n=73) and defects repaired with a patch under C (n=104). We are aware that this is somewhat arbitrary and not always true; e.g., sometimes a size B defect is repaired primarily and ECMO treatment can result in a small defect repaired with a patch.

Statistical analysis

IBM SPSS Statistics (version 27) was used for statistical analysis. To compare the categorical data between the groups a chi^2 test was used. A P-value of <0.05 was considered as statistically significant. Proportions within a group comparison were compared using an adjusted p-value (Bonferroni correction).

Table S1 Cohort description and statistics

		Sex			Associated anomalies			Defect size					Location of defect					
**Group**	**Characteristic**	**F**	**M**	**O**	**CDH-C**	**CDH-I**	**CDH-MD**	**A**	**B**	**C**	**D**	**MD**	**BL**	**EV**	**Left**	**POE**	**Right**	**MD**
**Associated anomalies**	**CDH-C (n=311)**	132[a] (42.4 %)	177[a] (56.9 %)	2[a] (0.6 %)	.	.	.	36[a] (11.6%)	15[a] (4.8%)	52[a] (16.7%)	11[a] (3.5%)	**197[a] (63.3%)**	6[a] (1.9%)	8[a] (2.6%)	228[a] (73.3%)	2[a] (0.6%)	54[a] (17.4%)	**13[a] (4.2%)**
	**CDH-I (n=475)**	214[a] (45.1 %)	252[a] (53.1 %)	9[a] (1.9 %)	.	.	.	80[a] (16.8%)	37[a] (7.8%)	112[a] (23.6%)	19[a] (4.0%)	**227[b] (47.8%)**	4[a] (0.8%)	10[a] (2.1%)	374[a] (78.7%)	4[a] (0.8%)	75[a] (15.8%)	**8[a] (1.7%)**
	**CDH-MD (n=19)**	12[a] (63.2 %)	6[a] (31.6 %)	1[a] (5.3 %)	.	.	.	0[a] (0.0%)	0[a] (0.0%)	5[a] (26.3%)	2[a] (10.5%)	**12[a, b] (63.2%)**	0[a] (0.0%)	0[a] (0.0%)	12[a] (63.2%)	0[a] (0.0%)	2[a] (10.5%)	**5[b] (26.3%)** *
	**Total (n)**	358 (44.5 %)	435 (54.0 %)	12 (1.5 %)	.	.	.	116 (14.4%)	52 (6.5%)	169 (21.0%)	32 (4.0%)	**436 (54.2%)**	10 (1.2%)	18 (2.2%)	614 (76.3%)	6 (0.7%)	131 (16.3%)	26 (3.2%)
	**P**	0.092			.	.	.	0.001229					0.000014					
**Defect size**	**A (n=116)**	43[a] (37.1 %)	73[a] (62.9 %)	0[a] (0.0 %)	**36[a, b] (31.0%)**	**80[a] (69.0%)**	0[a] (0.0%)	.	.	.	.	.	0[a] (0.0%)	**11[a] (9.5%)**	95[a] (81.9%)	2[a] (1.7%)	**8[a] (6.9%)**	**0[a, b] (0.0%)***
	**B (n=52)**	24[a] (46.2 %)	28[a] (53.8 %)	0[a] (0.0 %)	**15[a, b] (28.8%)**	**37[a, b] (71.2%)**	0[a] (0.0%)	.	.	.	.	.	0[a] (0.0%)	**1[a, b] (1.9%)***	44[a] (84.6%)	0[a] (0.0%)	**7[a, b] (13.5%)***	**0[a, b] (0.0%)***
	**C (n=169)**	79[a] (46.7 %)	90[a] (53.3 %)	0[a] (0.0 %)	**52[b] (30.8%)**	**112[a] (66.3%)**	5[a] (3.0%)	.	.	.	.	.	0[a] (0.0%)	**0[b] (0.0%)***	139[a] (82.2%)	2[a] (1.2%)	**28[a, b] (16.6%)***	**0[b] (0.0%)***
	**D (n=32)**	14[a] (43.8 %)	18[a] (56.3 %)	0[a] (0.0 %)	**11[a, b] (34.4%)**	**19[a, b] (59.4%)**	2[a] (6.3%)	.	.	.	.	.	0[a] (0.0%)	**0[a, b] (0.0%)***	25[a] (78.1%)	0[a] (0.0%)	**7[a, b] (21.9%)***	**0[a, b] (0.0%)***
	**MD (n=436)**	198[a] (45.4 %)	226[a] (51.8 %)	12[a] (2.8 %)	**197[a] (45.2%)**	**227[b] (52.1%)**	12[a] (2.8%)	.	.	.	.	.	10[a] (2.3%)	**6[b] (1.4%)***	311[a] (71.3%)	2[a] (0.5%)	**81[b] (18.6%)***	**26[a] (6.0%)**
	**Total (n)**	358 (44.5 %)	435 (54.0 %)	12 (1.5 %)	**311 (38.6%)**	**475 (59.0%)**	19 (2.4%)	.	.	.	.	.	10 (1.2%)	18 (2.2%)	614 (76.3%)	6 (0.7%)	131 (16.3%)	26 (3.2%)
	**P**	0.092			0.001229								7,24E-05					
**Location of defect**	**BL (n=10)**	3[a] (30.0 %)	7[a] (70.0 %)	0[a] (0.0 %)	6[a] (60.0%)	**4[a, b] (40.0%)**	**0[a, b] (0.0%)**	**0[a, b] (0.0%)**	0[a] (0.0%)	**0[a, b] (0.0%)**	0[a] (0.0%)	**10[a, b] (100.0%)**	.	.	.	.	.	.
	**EV(n=18)**	8[a] (44.4 %)	10[a] (55.6 %)	0[a] (0.0 %)	8[a] (44.4%)	**10[a, b] (55.6%)**	**0[a, b] (0.0%)**	**11[c] (61.1%)**	1[a] (5.6%)	**0[a, b] (0.0%)**	0[a] (0.0%)	**6[c] (33.3%)**	.	.	.	.	.	.
	**Left (n=614)**	280[a] (45.6 %)	326[a] (53.1 %)	8[a] (1.3 %)	228[a] (37.1%)	**374b (60.9%)**	**12[b] (2.0%)**	**95[a, b] (15.5%)**	44[a] (7.2%)	**139[a, b] (22.6%)**	25[a] (4.1%)	**311[c] (50.7%)**	.	.	.	.	.	.

	**POE (n=6)**	3[a] (50.0 %)	3[a] (50.0 %)	0[a] (0.0 %)	2[a] (33.3%)	**4[a, b] (66.7%)**	**0[a, b] (0.0%)**	**2[b, c] (33.3%)**	0[a] (0.0%)	**2[b] (33.3%)**	0[a] (0.0%)	**2[c] (33.3%)**	.	.	.	.	.	.
	**Right(n=131)**	50[a] (38.2 %)	77[a] (58.8 %)	4[a] (3.1 %)	54[a] (41.2%)	**75[a, b] (57.3%)**	**2[b] (1.5%)**	**8[a, b] (6.1%)**	7[a] (5.3%)	**28[a, b] (21.4%)**	7[a] (5.3%)	**81[b, c] (61.8%)**	.	.	.	.	.	.
	**MD (n=26)**	14[a] (53.8 %)	12[a] (46.2 %)	0[a] (0.0 %)	13[a] (50.0%)	**8[a] (30.8%)**	**5[a] (19.2%)**	**0[a] (0.0%)**	0[a] (0.0%)	**0[a] (0.0%)**	0[a] (0.0%)	**26[a] (100.0%)**	.	.	.	.	.	.
	**Total (n)**	358 (44.5 %)	435 (54.0 %)	12 (1.5 %)	311 (38.6%)	**475 (59.0%)**	**19 (2.4%)**	**116 (14.4%)**	52 (6.5%)	**169 (21.0%)**	32 (4.0%)	**436 (54.2%)**	.	.	.	.	.	.
	**P**	0.725			0.000014			7,24E-05										
	**F (n=358)**	.	.	.	132[a] (36.9%)	214[a] (59.8%)	12[a] (3.4%)	43[a] (12.0%)	24[a] (6.7%)	79[a] (22.1%)	14[a] (3.9%)	198[a] (55.3%)	3[a] (0.8%)	8[a] (2.2%)	280[a] (78.2%)	3[a] (0.8%)	50[a] (14.0%)	14[a] (3.9%)
	**M (n=435)**	.	.	.	177[a] (40.7%)	252a (57.9%)	6[a] (1.4%)	73[a] (16.8%)	28[a] (6.4%)	90[a] (20.7%)	18[a] (4.1%)	226[a] (52.0%)	7[a] (1.6%)	10[a] (2.3%)	326[a] (74.9%)	3[a] (0.7%)	77[a] (17.7%)	12[a] (2.8%)
**Sex**	**O (n=12)**	.	.	.	2[a] (16.7%)	9[a] (75.0%)	1[a] (8.3%)	0[a] (0.0%)	0[a] (0.0%)	0[a] (0.0%)	0[a] (0.0%)	12[b] (100.0%)	0[a] (0.0%)	0[a] (0.0%)	8[a] (66.7%)	0[a] (0.0%)	4[a] (33.3%)	0[a] (0.0%)
	**Total (n)**	.	.	.	311 (38.6%)	475 (59.0%)	19 (2.4%)	116 (14.4%)	52 (6.5%)	169 (21.0%)	32 (4.0%)	436 (54.2%)	10 (1.2%)	18 (2.2%)	614 (76.3%)	6 (0.7%)	131 (16.3%)	26 (3.2%)
	**P**				0.092			0.081					0.725					

Table S1 Cohort description and statistics. In total 805 patients were included in this cohort and there are more males (n=435, 54%) compared to females (n=358). Of 12 patient sex was not registered (1.5%). CDH is the only major anatomical malformation in the majority of our patients (Isolated-CDH; 73.3%). In the remaining 26.7% other associated anatomical anomalies were identified (Complex-CDH). Associated anomalies are not described in 475 patients of our cohort (isolated-CDH, 59%), 311 patients have one or more major structural anomalies (complex CDH, 38.6%) and of 19 patients there was no information about associated anomalies (2.4%). Defect location was not described in 26 patients (3.2%). Most hernias were left sided (n=614, 76.3%). In the remaining patients the herniation was on the right side of the diaphragm (16.3%, 131 patients), six were diagnosed with a congenital para-esophageal hernia (0.7%), ten patients with a bilateral herniation (1.2%) and 18 patients with eventration of the diaphragm (2.2%). Defect size (A-D) was described in 368 patients (n=45.7%). Primarily repaired defects under without a classification were assigned under A (n=73) and defects repaired using a patch under C (n=104). Within a row each outcome measure that does not share a subscript letter ([a, b, c]) differs significantly from those with different subscript letters ([a, b, c]) whose column proportions do not differ significantly from each other at the .05 level. Significant differences in outcome measure subgroups can be assigned to missing data. For instance, the group "missing data" differs from complex and isolated CDH (0.001229) when looking at defect size as well as defect location (p=0.000014). Defect size C differs from the missing data category in complex CDH and isolated-CDH (p= 0.001229). Right sided hernia and missing data differ when comparing defect sizes (p=7,24E-05). Registration an classification of repair is responsible of the differences in the eventration category. These were assigned a defect size of A (n=11), B (n=1) or were not repaied (n=5). MD; Missing data, CDH-C; CDH patients with associated defects, CDH-I; CDH patients without other associated defects, CDH-MD; CDH patients in which no additional information was registered, POE; Paraoesophageal hernia, EV; Eventration, BL; Bilateral hernia, AGT; abnormal genetic test, NPC; no pathogenic changes

Table S2 Cohort description: genetics and statistics

		Location of defect						Associated anomalies			Sex			Result	
**Group**	**Characteristic**	**BL (n=4)**	**EV (n=17)**	**Left (n=415)**	**POE (n=4)**	**Right (n=73)**	**MD (n=17)**	**CDH-C (n=207)**	**CDH-I (n=311)**	**CDH-MD (n=12)**	**F (n=238)**	**M (n=285)**	**O (n=7)**	**AGT (n=62)**	**NPC (n=468)**
**Sex**	**F**	2[a] (50.0%)	8[a] (47.1%)	186[a] (44.8%)	1[a] (25.0%)	31[a] (42.5%)	10[a] (58.8%)	95[a] (45.9%)	135[a] (43.4%)	8[a] (66.7%)	-	-	-	34[a] (14.3%)	204[a] (85.7%)
	**M**	2[a] (50.0%)	9[a] (52.9%)	225[a] (54.2%)	3[a] (75.0%)	39[a] (53.4%)	7[a] (41.2%)	111[a] (53.6%)	170[a] (54.7%)	4[a] (33.3%)	-	-	-	27[a] (9.5%)	258[a] (90.5%)
	**O**	0[a] (0.0%)	0[a] (0.0%)	4[a] (1.0%)	0[a] (0.0%)	3[a] (4.1%)	0[a] (0.0%)	1[a] (0.5%)	6[a] (1.9%)	0[a] (0.0%)	-	-	-	1[a] (14.3%)	6[a] (85.7%)
	**P**	0.693						0.334						0.228	
**Location of defect**	**BL (n=4)**	-	-	-	-	-	-	1[a] (0.5%)	3[a] (1.0%)	0[a] (0.0%)	-	-	-	0[a] (0.0%)	4[a] (100.0%)
	**EV (n=17)**	-	-	-	-	-	-	7[a] (3.4%)	10[a] (3.2%)	0[a] (0.0%)	-	-	-	1[a] (5.9%)	16[a] (94.1%)
	**Left (n=415)**	-	-	-	-	-	-	159[a] (76.8%)	246[a] (79.1%)	10[a] (83.3%)	-	-	-	48[a] (11.6%)	367[a] (88.4%)
	**POE (n=4)**	-	-	-	-	-	-	2[a] (1.0%)	2[a] (0.6%)	0[a] (0.0%)	-	-	-	2[a] (50.0%)	2[a] (50.0%)
	**Right (n=73)**	-	-	-	-	-	-	29[a] (14.0%)	43[a] (13.8%)	1[a] (8.3%)	-	-	-	7[a] (9.6%)	66[a] (90.4%)
	**MD (n=17)**	-	-	-	-	-	-	9[a] (4.3%)	7[a] (2.3%)	1[a] (8.3%)	-	-	-	4[a] (23.5%)	13[a] (76.5%)
	**P**							0.936						0.094	
**Associated anomalies**	**CDH-C (n=207)**	1[a] (25.0%)	7[a] (41.2%)	159[a] (38.3%)	2[a] (50.0%)	29[a] (39.7%)	9[a] (52.9%)	-	-	-	-	-	-	56[a] (27.1%)	151[a] (72.9%)
	**CDH-I (n=311)**	3[a] (75.0%)	10[a] (58.8%)	246[a] (59.3%)	2[a] (50.0%)	43[a] (58.9%)	7[a] (41.2%)	-	-	-	-	-	-	6[b] (1.9%)	305[b] (98.1%)
	**CDH-MD (n=12)**	0[a] (0.0%)	0[a] (0.0%)	10[a] (2.4%)	0[a] (0.0%)	1[a] (1.4%)	1[a] (5.9%)	-	-	-	-	-	-	0[a, b] (0.0%)	12[a, b] (100.0%)
		0.936												1,43E-14	
**Defect size**	**A (n=97)**	0[a, b, c] (0.0%)	11[c] (64.7%)	78[a, b] (18.8%)	2[b, c] (50.0%)	6[a, b] (8.2%)	0[a] (0.0%)	31[a] (15.0%)	66[a] (21.2%)	0[a] (0.0%)	34[a] (35.1%)	63[a] (64.9%)	0[a] (0.0%)	10[a, b] (10.3%)	87[a, b] (89.7%)
	**B (n=50)**	0[a] (0.0%)	1[a] (5.9%)	42[a] (10.1%)	0[a] (0.0%)	7[a] (9.6%)	0[a] (0.0%)	15[a] (7.2%)	35[a] (11.3%)	0[a] (0.0%)	24[a] (48.0%)	26[a, b] (52.0%)	0[a] (0.0%)	4[a, b] (8.0%)	46[a, b] (92.0%)
	**C (n=157)**	0[a, b, c] (0.0%)	0[c] (0.0%)	132[a, b, c] (31.8%)	2[b] (50.0%)	23[a, b, c] (31.5%)	0[a, c] (0.0%)	46[a] (22.2%)	106[b] (34.1%)	5[a, b] (41.7%)	71[a] (45.2%)	86[a, b] (54.8%)	0[a] (0.0%)	5[b] (3.2%)	152[b] (96.8%)
	**D (n=32)**	0[a] (0.0%)	0[a] (0.0%)	25[a] (6.0%)	0[a] (0.0%)	7[a] (9.6%)	0[a] (0.0%)	11[a] (5.3%)	19[a] (6.1%)	2[a] (16.7%)	14[a] (43.8%)	18[a, b] (56.3%)	0[a] (0.0%)	2[a, b] (6.3%)	30[a, b] (93.8%)
	**MD (n=194)**	4[a, b] (100.0%)	5[b] (29.4%)	138[b] (33.3%)	0[b] (0.0%)	30[b] (41.1%)	17[a] (100.0%)	104[a] (50.2%)	85[b] (27.3%)	5[a, b] (41.7%)	95[a] (49.0%)	92[b] (47.4%)	7[a] (3.6%)	41[a] (21.1%)	153[a] (78.9%)
	**P**	1,84E-04						0.000027			0.016			0.000006	

CDH heritability

S2 Cohort description: genetics and statistics: In total, 530 out of 805 patients received a genetic test. Defect size (A-D) was described in 336 patients. Defect sizes are classified from A to D as described in the method section. A is the smallest defect size and D a (near) absence of the diaphragm. Within a column each outcome measure that does not share a subscript letter ($^{a, b, c}$) differs significantly from those with different subscript letters ($^{a, b, c}$) whose column proportions do not differ significantly from each other at the .05 level. For instance, more patients with associated anomalies have an abnormal test and vice versa more patients with an isolated defect have no abnormal test (p=1,432E-14). Patients with defect size C have differ in the number of abnormal tests to the missing data group (P=0.000006). Trisomy 13,18 and 21 were evaluated in 530 patients and more than half of the patients received at least karyotyping or SNP-array. MD; Missing data, CDH-C; CDH patients with associated defects, CDH-I; CDH patients without other associated defects, CDH-MD; CDH patients in which no additional information was registered, POE; Paraoesophageal hernia, EV; Eventration, BL; Bilateral hernia, AGT; abnormal genetic test, NPC; no pathogenic changes.

S3 Exome sequencing coding variation differences between discordant monozygotic twins

Methods

Discordant monozygotic twin pairs were selected from the Erasmus University MC-Sophia Children's hospital cohort. DNA was extracted from peripheral blood when the twins were at least one year of age to avoid contamination with sibling DNA resulting from transfer of lymphocytes via twin-to-twin transfusion. Unfortunately, transfer of hematopoietic stem cells can't be excluded[3]. We used DNA derived from skin fibroblasts in CDH patients CDH-01 and CDH-02. Monozygosity was determined with short tandem repeat profiling (AmpFISTR identifier PCR amplification kit, Applied Biosystems, Foster City, CA, USA) and later confirmed with SNP-array. Patients did not have confirmed genetic syndromes or *de novo* chromosomal anomalies prior to analysis. Exome capture (SureSelect Human All Exon 50 Mb Targeted exome enrichment kit v2, Agilent Technologies, Inc., Santa Clara, California), paired end sequencing (Illumina, Inc., San Diego, USA), alignment to the hg19 reference genome and filtering strategies are described previously [4].

Results

Twin-Pair	GA	TAR	TART	ACT	20X	GATK	NBS	FRF	SSV
**CDH-01**	35.3	71997905-96482865	51756113-51756099	58.22-82.13	82.4-87.1	284	27	102	0
**CDH-02**	33.4	82217707-80667781	51755869-51756015	85.43-87.26	87.1-88.8	200	28	1	0
**CDH-03**	38.5	49504665-80667781	51753042-51755968	52.53-73.42	73.6-86.7	117	2	1	0
**CDH-04**	34.1	72176954-80549115	51756122-51756078	65.65-76.47	84.7-86.8	145	15	23	0

CDH heritability

CDH affected twins all have Congenital Diaphragmatic Hernia and siblings are healthy. Depicted are the differences between affected-unaffected siblings per twin pair. CDH-01 and CDH-02 had isolated CDH. CDH-03 (Urogenital malformations, inguinal hernia and hydrocele testis) and CDH-04 (Dysmorphic features, small mandibula and intrauterine growth restriction) were complex CDH patients. All DNA was derived from peripheral blood, except for patient CDH-01 and CDH-02 (DNA derived from dermal fibroblasts). GA; Gestational age in weeks. TAR; total of aligning reads, TART; total aligning reads on target (the "exome" captured), ACT; average coverage on target, 20X; percentage of target covered ≥ 20X read depth, GATK; GATK-unified Genotype, NBS; Negative Binomial Statistics, FRF; Fisher exact + Repeat Filter, SSV; Sanger sequencing validation. in GATK, NBS, FRF and SSV are those after filtering and include all exonic, noncoding exonic and putative splicing variants ≥ 20X with minor allele frequency below 0.001.

References

[1] K. Tsao, and K.P. Lally, The Congenital Diaphragmatic Hernia Study Group: a voluntary international registry. Semin Pediatr Surg 17 (2008) 90-7.

[2] K.P. Lally, R.E. Lasky, P.A. Lally, P. Bagolan, C.F. Davis, B.P. Frenckner, R.M. Hirschl, M.R. Langham, T.L. Buchmiller, N. Usui, D. Tibboel, and J.M. Wilson, Standardized reporting for congenital diaphragmatic hernia--an international consensus. Journal of pediatric surgery 48 (2013) 2408-15.

[3] Y. Erlich, Blood ties: chimerism can mask twin discordance in high-throughput sequencing. Twin research and human genetics : the official journal of the International Society for Twin Studies 14 (2011) 137-43.

[4] R.W. Brouwer, M.C. van den Hout, F.G. Grosveld, and W.F. van Ijcken, NARWHAL, a primary analysis pipeline for NGS data. Bioinformatics (Oxford, England) 28 (2012) 284-5.
